# Children having children: early motherhood and offspring human capital in India

**DOI:** 10.1007/s00148-023-00946-0

**Published:** 2023-03-28

**Authors:** M. Perez-Alvarez, M. Favara

**Affiliations:** 1 University of Groningen, Faculty of Economics and Business, Universiteitscomplex, 9747 Groningen, AJ Netherlands; 2 University of Oxford, Department of International Development, 3 Mansfield Road, Oxford, OX1 3TB UK

**Keywords:** I15, I25, J13, J16, O15, Early motherhood, Fertility, Health, Cognition, Human capital, Gender

## Abstract

Using panel data from India, this paper investigates the effect of early maternal age on offspring human capital, contributing to the scarce evidence on this phenomenon, especially in the context of a developing country. The analysis relies on mother fixed effects to allow for unobserved differences between mothers and employs a variety of empirical strategies to address remaining sibling-specific concerns. Our results indicate that children born to young mothers are shorter for their age, with stronger effects for girls born to very young mothers. We also find some evidence suggesting that children born to very young mothers perform worse in math. By exploring the evolution of effects over time for the first time in the literature, we find that the height effect weakens as children age. Further analysis suggests both biological and behavioral factors as transmission channels.

**Supplementary Information:**

The online version contains supplementary material available at 10.1007/s00148-023-00946-0.

## Introduction

Early motherhood remains a widespread phenomenon in low- and middle-income countries (LMICs), where approximately 18 million adolescent girls give birth every year, amounting to 95% of global adolescent births (EWEC 2015; UNFPA 2015; WHO 2014; Neal et al. 2012).^1^ Such scale of early motherhood might have important implications for offspring development in LMICs. For instance, the medical literature has linked early pregnancy to labor complications and poor neonatal outcomes (Neal et al. 2018; Fall et al. 2015; Gibbs et al. 2012; Neal et al. 2012; Conde-Agudelo et al. 2005). In the same vein, the important role that mothers play in their children’s human capital acquisition suggests that mother’s age and its associated knowledge, bargaining power, and overall independence might be crucial for child development (Doss 2013; Duflo 2003). Such medical and behavioral implications of early maternal ages gain further relevance in light of the long-term consequences of prenatal and early childhood circumstances (e.g., Almond et al. 2018; Comfort 2016; Almond and Mazumder 2011).

In this paper, we investigate the causal effect of early motherhood on offspring human capital, both in terms of health and cognition, in the Indian states of Andhra Pradesh and Telangana, where 12% and 11%, respectively, of females aged 15–19 had already had a live birth or were pregnant at the time of data collection (IIPS 2017). Our analysis is guided by the Indian legal framework, which sets the legal age of sexual consent, as well as the legal independence of individuals, at 18 years of age. Importantly, while this age cut-off is anchored in the Indian Penal Code, motherhood at younger ages is not uncommon (Criminal Law Act 2013). Our analysis sheds light on whether the violation of this law bears intergenerational consequences affecting the human capital of children born to adolescent mothers, defined as mothers under age 18.

In addition, we investigate for the first time how the early motherhood effect evolves over time, covering the offspring transition from childhood into early adolescence. Furthermore, we test for heterogeneous effects across disaggregated groups of early motherhood and offspring gender and explore potential transmission channels.

The main empirical challenge for our purpose is unobserved mother and family characteristics. As women from poor socioeconomic backgrounds are more likely to experience early motherhood, differences in offspring outcomes by maternal ages may simply reflect differences in prechildbearing characteristics.^2^ Moreover, mother cohort effects might confound the effect of interest. Using longitudinal data spanning 7 years on 1690 sibling pairs, we circumvent these issues by adopting a mother fixed effects approach (MFE). We thereby exploit the maternal age at birth variation within the same family and compare offspring outcomes of children born to the same biological mother.^3^ To ease remaining concerns related to sibling unobserved heterogeneity, we perform the Oster (2019) method, run falsification tests with varying maternal age cut-offs, investigate the older sibling effect, and control for household-specific socioeconomic progress over time.

Our estimates suggest that early maternal age is detrimental to offspring health. We find that being born to an adolescent mother is associated with 0.23 lower height-for-age z-scores (HAZ), compared with children born to adult mothers. The effect is largest at early ages and weakens as children enter adolescence. This points to a partial catch-up over time, in line with childhood growth dynamics suggested by Anand et al. (2018) and Desmond and Casale (2017). In spite of this trajectory, effects in early adolescence are more than 2.4 times larger than estimates from a developed country context (Aizer et al. forthcoming). What is more, as physical growth is minimal after early adolescence, this finding implies that the detrimental effect is permanent in the offspring lives. Furthermore, the magnitude of the point estimate increases for children born to very young mothers (aged < 16) and is even stronger for their female offspring, consistent with previous evidence on gender discrimination in parental human capital investments in India as a response to adverse circumstances (Asfaw et al. 2010; Rose 1999; Behrman 1988).

In our cognition analysis, we find no effect for children born to adolescent mothers and a detrimental cognition effect for children born to very young mothers. These children perform 0.31 SD lower in the math test in their early adolescence, a point estimate that is 1.7 times larger than cognition effects found for the Norwegian context (Aizer et al. forthcoming).^4^ The magnitude of the effect is again larger among girls. Also, the effect surges at later ages, in line with the self-productivity rationale of cognitive development suggested by Cunha et al. (2006) according to which skill attainment at one stage of the life cycle raises skill attainment at later stages.

Turning to the transmission channel analysis, we find suggestive evidence on the role of birthweight, dietary diversity, and maternal involvement in education as three mechanisms explaining the negative relationship between early maternal age and child human capital. The birthweight analysis speaks closely to biological channels, although behavioral aspects during pregnancy might also be at play. The results on dietary diversity and maternal involvement in education are in turn indicative of behavioral mechanisms.

We use a variety of empirical strategies to address remaining endogeneity concerns that are inherent to sibling-difference models, two of which we summarize now. First, we observe supporting evidence from the Oster method, which indicates that for the estimated early motherhood effects to be null, unobserved sibling-specific factors would have to be more important for selection than age, gender, birth order, and all household and mother-level factors shared by siblings, which we believe to be improbable.

Furthermore, the falsification tests for HAZ fail to reproduce our main results. As these tests essentially emulate the comparison of siblings from our main specification with the difference of applying higher maternal age cut-offs located in adulthood, the absence of a negative association suggests that inherent aspects of sibling comparisons that are unrelated to early motherhood do not drive our results.^5^

Our analysis contributes to the economic literature on the long-term consequences of prenatal and childhood environments on human development (e.g., Almond et al. 2018; Comfort 2016; Almond and Mazumder 2011). We posit mother’s age at the time of birth as a relevant factor influencing both the prenatal and postnatal environment of children, affecting their human capital development and ultimately adult outcomes. Importantly, we contribute to this literature by covering the offspring transition from childhood into adolescence, a period recently referred to as the missing middle, given the scarcity of studies on this key developmental stage. Expanding our knowledge on this stage is of particular relevance for the feasibility of policy interventions after early childhood (Almond et al. 2018).

Furthermore, we contribute to the literature on the consequences of early motherhood in several ways. First, this paper significantly advances the literature by using data from LMICs. Such studies are remarkably scarce despite the concentration of global adolescent births in LMICs. Besides this geographical concentration, investigating adolescent motherhood in the context of LMICs provides important insights, as resources to counteract detrimental effects are likely to be scarce. In this respect, the magnitude of early motherhood consequences and its persistence over time might crucially depend on factors that vary across economic development stages, such as institutional and family safety nets. For instance, resources to counteract detrimental effects are likely scarcer in LMICs settings, particularly among poor communities with poor access to robust safety-nets. To the best of our knowledge, Branson et al. (2015) is the only existing study investigating the effect of maternal age on offspring development in a LMIC context.^6^ Using propensity score matching to analyze data from Cape Town, South Africa, the authors find that children born to early mothers are shorter for their age and have lower birthweight. Moreover, we make an important contribution by addressing endogeneity with a sibling-difference framework in a LMIC context for the first time. This approach is an established tool in the literature on human capital production and in particular among early motherhood studies using data from high-income countries.

Interestingly, the evidence from high-income countries is mixed. While earlier studies conclude that observed offspring differences in birthweight and cognitive skills, among others, are the result of unobserved prechildbearing characteristics (Levine et al. 2007; López-Turley 2003; Rosenzweig and Wolpin 1995; Geronimus et al. 1994), more recent ones suggest that early motherhood is indeed detrimental to offspring development indicators in young adulthood such as height and cognitive scores (Aizer et al. forthcoming; Carslake et al. 2017), non-cognitive skills (Carslake et al. 2017) and educational attainment, and income (Aizer et al. forthcoming; Francesconi 2008).

Finally, we make a significant contribution to the literature by exploiting the panel dimension of the data and exploring for the first time the evolution of the early motherhood effect over time, which allows us to obtain a wider perspective of the association of interest and to cover an under-studied human development stage as previously discussed.

The rest of the paper unfolds as follows. Section 2 describes the data used. Section 3 outlines the empirical strategy. Section 4 presents the main results, dynamics over time, heterogeneous effects and transmission channels. Section 5 concludes.

## Data and descriptive statistics

We use household data from the Young Lives study for our analysis. Young Lives is a longitudinal study on childhood poverty following 12,000 children of two cohorts in Ethiopia, India (Andhra Pradesh and Telangana), Peru, and Vietnam over 15 years. The older cohort consists of around 1000 children per country who were born in 1994–1995 and tracked since ~age 8, while the younger cohort of around 2000 children per country was born in 2001–2002 and tracked since ~age 1. We restrict our analysis to the younger cohort data from India, given the prevalence of early motherhood in the sample and the availability of sibling data. The first study round was in 2002, when the children were 1 year old. It was followed by four subsequent rounds in 2006 (age 5), 2009 (age 8), 2013 (age 12), and 2016 (age 15).

The sampling design consisted of two stages. In the first stage, 20 clusters (mandals) were sampled based on a set of economic, human development, and infrastructure indicators with the purpose of oversampling poor households. Hence, the Young Lives household surveys do not constitute a nationally representative survey, although it does cover the diversity of children in the country (Young Lives 2017; Kumra 2008). In the second stage, approximately 100 households with a child born in 2001–2002 were randomly selected from each cluster. The initial sample for the younger cohort in India consisted of 2011 children living both in rural and urban communities and spread across seven districts in three regions.^7^ These children are referred to as index children in this paper. The attrition rate across all five rounds is only 6%, a remarkably low value considering the time period covered by the study.

Since the third survey round in 2009, additional anthropometric (rounds 3 to 5) and cognition (rounds 4 and 5) data were collected on one sibling of each index child. Among available siblings, the next younger sibling of the index child was selected. If not available, the next older sibling was interviewed.^8^ For the current analysis, we restrict our sample to sibling pairs, composed of the index child and a younger or older sibling, with available data on height-for-age (HAZ) or math performance and the relevant child-level control variables used in the empirical analysis.^9^ We end up using observations from 1690 households with sibling pairs, of which 910 contain a younger sibling and 754 an older one.^10^ The age gap between panel siblings and index children is remarkably symmetric. Older siblings are on average 3 years older, while younger siblings are 3 years younger on average.^11^ In our sample, all sibling pairs are reported to have the same biological parents. Note that the time period of our sample covers the transition of children from middle childhood to adolescence, a phase in child development that is understudied (Almond et al. 2018).

**Fig. 1:**
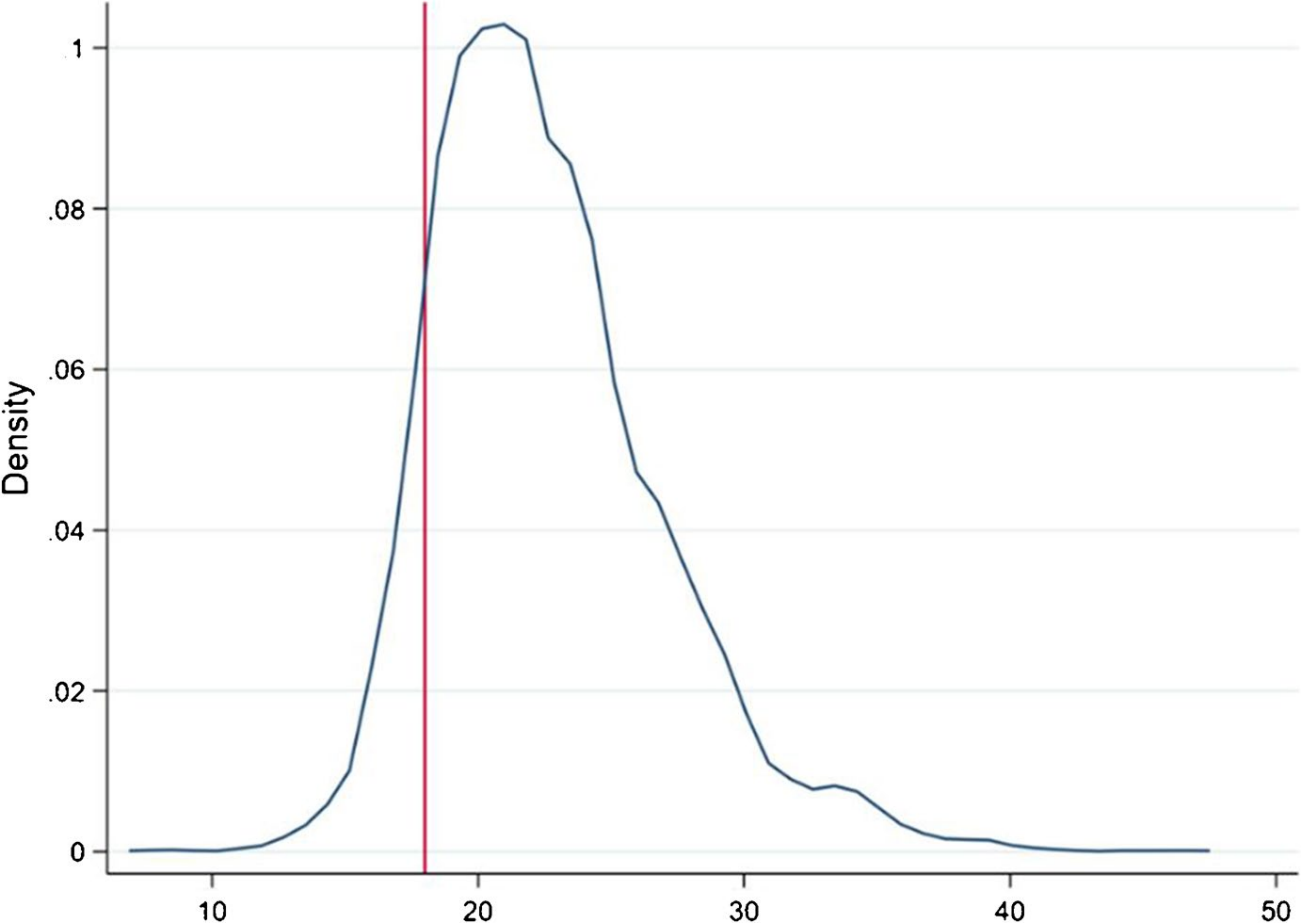
Kernel density of maternal age at birth. Notes: Maternal ages at birth for all sibling pairs used in the main empirical analysis. Red line indicates cut-off of 18 years of age. Kernel (epanechnikov) estimation with bandwith of 0.7080

In this paper, maternal age at birth is constructed as the difference between the child’s age and mother’s age.^12^ Figure 1 shows the distribution of maternal age at birth for the sibling pairs used in the main empirical analysis. The average maternal age is 23 years and the distribution is quite dispersed. For the empirical analysis, we use these values to compute binary indicators for children born to adolescent mothers (aged < 18), to young mothers (16–17), and to very young mothers (< 16).^13^

Besides anthropometric and cognition data, we obtain information on the geographic location of the household (the state and mandals of residency and whether the household is in a rural/urban area) and on the socioeconomic background of the children as indicated by maternal education in terms of highest grade completed, total expenditure of the household in real terms, a wealth index, which consists of a composite measure of living standards (see Briones (2017) for details), and mother’s height, as an indicator of potential intergenerational cycles of health deficits and poverty. Moreover, we observe the ethnicity of children, as well as their gender, age, and birth order. The latter is constructed by comparing the ages of all the siblings living in the same household during any of the survey rounds.

Table 1 shows the sample average characteristics of the pairs of siblings born to adult mothers (80.77%), the “switchers” with one of the siblings born to an adolescent mother and the other one born when she was an adult (17.28%), and the pairs of siblings both born during adolescence (1.95%). Note that MFE models exploit the presence of switchers in the sample. As expected, sibling pairs with one and two children born to adolescent mothers come from families with poorer socioeconomic background than children born to adult mothers. Their mothers have lower education, tend to be shorter, live in households that have lower total expenditures per capita and are less wealthy. Furthermore, these pairs are more likely to live in rural areas and to be a member of a disadvantaged ethnicity/caste than the pairs with both siblings born to adult mothers. In line with this pattern, the differences are stronger when looking at pairs with both siblings born to adolescent mothers.^14^

**Table 1:** Sample characteristics by maternal age group

Sibling pair	Both born to adult mother (age > 18)	One born to an adolescent mother (age < 18)	Both born to adolescent mother (age < 18)
	Mean	Mean	Mean
Household characteristics			
Maternal age at birth	23.62	18.44	16.19
Mother's education	3.04	2.69	2.39
Mother's height	151.69	150.59	148.57
Total expenditure	958.92	882.27	841.11
Wealth tertiles			
First wealth tertile	0.55	0.60	0.70
Second wealth tertile	0.25	0.31	0.27
Third wealth tertile	0.20	0.10	0.03
Region			
Urban	0.26	0.21	0.09
Coastal Andrah	0.33	0.39	0.30
Rayalaseema	0.31	0.27	0.21
Telangana	0.36	0.34	0.48
Ethnicity/caste			
Scheduled caste	0.17	0.21	0.21
Scheduled tribe	0.14	0.16	0.21
Backward class	0.46	0.50	0.45
Other	0.22	0.13	0.12
Older sibling characteristics			
Age	16.21	17.16	16.37
Female	0.51	0.46	0.55
First born	0.58	0.88	0.85
Second born	0.26	0.11	0.15
Third born	0.10	0.01	0.00
Very young mother (age<16)	0.00	0.17	0.64
Young mother (ages 16–17)	0.00	0.83	0.36
Younger sibling characteristics			
Age	13.28	13.61	14.42
Female	0.49	0.46	0.30
First born	0.00	0.00	0.00
Second born	0.58	0.85	0.88
Third born	0.26	0.13	0.09
Very young mother (age<16)	0.00	0.00	0.06
Young mother (ages 16–17)	0.00	0.00	0.94
Observations	1,365	292	33

Notes: Statistics in Table 1 correspond to sibling pair-level observations from the pooled sample of households with available information on age, gender, birth order, maternal age, and HAZ or math data for the sibling pairs participating in rounds 3 (2009), 4 (2013), and 5 (2016). Time-variant variables such as wealth tertiles, total expenditure, location-related variables, and mother’s education uses baseline values, and age uses round 5 values. Maternal age is computed by averaging differences between child’s age and mother’s age across rounds. Mother’s education consists of highest completed grade. Mother’s height is reported in cm. Total expenditure refers to household total monthly expenditure per capita in 2006 constant rupees. A composite wealth index was used for the estimation of the share of observations within each wealth tertile (see Briones (2017) for a detailed description). For the computation of birth order, the ages among siblings that lived in the Young Lives household during any of the 5 survey rounds were compared.

These raw differences, suggesting socioeconomic disadvantages for children born to earlier mothers, manifest the empirical challenge of disentangling the effects of maternal age and socioeconomic background on offspring development and highlight the importance of mother fixed effects. Finally, Table 1 shows that older siblings are more likely to be older, to be a firstborn and to be born to a very young mother than younger siblings.

We use HAZ z-scores and math Item Response Theory (IRT) scores as our health and cognition outcomes, respectively Figure A1. HAZ is a universally comparable indicator of child growth standardized according to age- and gender-specific child growth references of a well-nourished population (WHO 2007).^15^ A deficit in a child’s HAZ is an indicator for chronic malnutrition and cumulative deficient growth widely used in economics (e.g., Larsen and Lilleør 2017; Liu 2014; Alderman 2000). Furthermore, it is less sensitive to temporary shocks related to morbidity, illnesses or seasonal variations in food availability than other nutritional indicators, such as weight-for-age and weight-for-height.

For the computation of math scores, the survey team developed a mathematics test, which was adapted for each survey round to ensure its appropriateness (Cueto and Leon 2012; Cueto et al. 2009). The math test was administered to all children, regardless of whether or not they were attending school at the time of the interview. It was not designed for a specific school grade but rather to incorporate questions at widely differing levels of difficulty. At the basic level, the tests included questions assessing basic number identification and quantity discrimination; at the intermediate level, questions were based on calculation and measurement; and at the advanced level, questions related to problem-solving embedded in hypothetical contexts that simulate real-life situations (e.g., tables in newspapers).

The test scores used in this paper are constructed using IRT models, which are commonly used in international assessments such as Programme for International Student Assessment (PISA) and Trends in International Mathematics and Science Study (TIMSS). The main advantages of IRT models consist of acknowledging item difficulty and enhancing comparability over time and across ages (see Leon and Singh (2017) for more details).

Table 2 presents the mean values of the outcomes of interest by maternal age groups.^16^ The mean values of HAZ across maternal age groups suggest a negative relationship between offspring health and early motherhood. While all groups show negative mean values, indicating that all children on average present growth deficits, children born to adolescent mothers show larger deficits than children born to adult mothers. Moreover, children born to very young mothers do worse than children born to young mothers. These raw differences are all statistically significant at the 1% level. Math scores show a different pattern. Children born to adolescent mothers perform better than children born to adult mothers when comparing unadjusted means. However, children born to very young mothers show worse values than those born to young mothers. The raw differences in math are all at least marginally statistically significant.

**Table 2:** Average outcomes by maternal age group

Outcome	HAZ			Math		
Born to	*Mean*	*SD*	*N*	*Mean*	*SD*	*N*
Very young mothers (age < 16)	−1.94	1.02	147	444.72	118.12	118
Young mothers (ages 16–17)	−1.72	1.03	749	484.56	98.04	497
Adolescent mothers (age < 18)	−1.75	1.03	896	476.91	103.29	615
Adult mothers (age ≥ 18)	−1.45	1.15	7,802	465.05	111.50	5,257
All mothers	−1.49	1.15	8698	466.30	110.73	5872

Notes: The statistics in Table 2 correspond to child-round-level observations from the pooled sample of households with available information on age, gender, birth order, maternal age, and the respective outcome variable for the sibling pairs participating in rounds 3 (2009), 4 (2013), and 5 (2016). Ages in parentheses refer to maternal ages at birth. HAZ is height-for-age in z-scores collected in the three rounds, while math consists of IRT scores collected in rounds 4 and 5. Mean differences across maternal age groups are statistically significant at least at the 5.1% level.

While the patterns in Table 2 are informative, it is plausible that the gaps across maternal age groups are a reflection of differences in the socioeconomic background of children, mother cohorts, family sizes, and/or their age, gender, and birth order profile, among others. We follow a regression framework, as described in the next section to adjust these raw differences in an attempt to isolate the main effect of interest.

## Empirical strategy

Our estimates of the impact of maternal age at birth on the health and cognition of children are nested in a theoretical framework that models the human capital production of children (Attanasio 2015; Cunha et al. 2006; Todd and Wolpin 2003).^17^ In this section, we describe the empirical approach used to overcome the main empirical challenges encountered in estimating the effect of early maternal age on offspring human capital. First, poorer outcomes of children born to adolescent mothers might be the result of unobserved disadvantaged socioeconomic background rather than the consequences of early motherhood itself. In this case, the adolescent motherhood effect would be overestimated.

Second, while adolescent mothers might have a poorer socioeconomic background in comparison to their peers, they grew up in a more recent time period than older mothers. In a context of general socioeconomic progress over time, women who grew up in say the 1990s rather than the 1970s were exposed to a relatively improved prenatal, postnatal, and childhood environment, for example, in terms of better health and education services. Neglecting these unobservables would downwardly bias the estimate of interest.

To tackle these sources of endogeneity, we exploit the availability of sibling data and the presence of switcher sibling pairs in our data. These are siblings that were born during different maternal age categories and thus show varying adolescent mother status, which allows us to rely on mother fixed effects (MFE). That is, we account for mother’s unobserved characteristics by looking at the outcomes of offspring pairs born to the same biological mother. The MFE estimates have the main advantage of accounting for all time-invariant mother and household-specific factors common to the index child and the panel sibling (including shared genetic factors and mother cohort effects), as well as for all unobserved context-specific factors that are constant among siblings (including access to health and education services). Moreover, these estimates account for differences in family sizes, which can affect offspring human capital in several ways (Spears et al. 2019; Behrman and Taubman 1986).^18^

Specifically, we estimate regression model (1) to investigate the relationship between adolescent motherhood and *Y*_*ijr*_ , which denotes a health or cognition outcome *Y* measured at round *r* for offspring *i* born to mother *j*. *AM*_*ij*_ is a dummy variable indicating children born to adolescent mothers, defined as mothers under 18 years of age at childbirth. The parameter of interest *β* identifies the effect of being born to an adolescent mother on child’s health or cognitive outcomes, compared to children born to adult mothers (18 years old or older). In a further specification, we distinguish between children born to very young mothers (under 16 years old) and those born to young mothers (16–17 years old).

1 $${Y}_{ij r}=\alpha +\beta {AM}_{ij}+\omega {Z}_{ij r}^{\prime }+{\mu}_j+{\theta}_r+{\varepsilon}_{ij r}$$

${Z}_{ijr}^{\prime }$ is a vector of child’s characteristics such as gender, age fixed effects, and birth order fixed effects; *μ*_*j*_ are the mother fixed effects; *θ*_*r*_ are data round fixed effects; and *ε*_*it*_ is an error term, clustered at the mother level to correct for within-family correlation.^19^ The two outcome variables are height-for-age z-scores (HAZ) and math IRT scores, collected for the sibling pairs in rounds 3, 4 and, 5 and rounds 4 and 5, respectively.

We also present OLS estimates for comparison purposes. For these estimates, we include additional prechildbearing controls at the mother level, such as ethnicity/caste fixed effects with categories for Scheduled caste, Scheduled tribe, Backward class, and Other, mother’s height (in cm), and rural/urban location of the household residence in round 1.^20^ Mother’s height is a good measure of maternal health and past nutrition, reflecting accumulated investments she has been exposed to during her (prechildbearing) lifetime and, to some extent, genetic predisposition (Subramanian et al. 2009; Duflo 2000). Furthermore, there might be a certain degree of intergenerational persistence in nutritional status which suggests that maternal nutrition might indeed be an important factor to explain child nutritional status (see for an example Ramakrishnan et al. (1999)). We abstain from including factors at the mother level that might be affected by childbearing in the OLS regressions, as they would constitute an endogenous control.

It is worth emphasizing that controlling for birth order fixed effects is relevant. We acknowledge that birth order might affect a child’s development for a number of reasons and in an a priori unknown direction (see De Haan et al. (2014) for a review of studies testing negative and positive birth order effects in developed and developing countries). For instance, children of higher birth order might either benefit from learning-by-doing parenting effects or be negatively affected by the relaxation of rearing practices over time (Lehmann et al. 2018).

Another example of the importance and ambiguity of birth order effects relates to financial resources. While one could argue that first-born children might benefit from exclusive expenditure in the first years of life and even longer-term parental favoritism, they might also be — to the detriment of their development — more exposed to child labor in comparison to their siblings (Jayachandran and Pande 2017; De Haan et al. 2014).

Importantly for our analysis, higher birth order has been shown to have a *negative* effect on height among children aged 0–5 years in India (Jayachandran and Pande, 2017), among children aged 6–10 years in Austria (Pruckner et al., 2021), and among adults in Sweden (Myrskylä et al., 2013). Since being born to adolescent mothers is associated with lower birth orders, this evidence suggests that failing to account for birth order effects would bias the adolescent motherhood effect upwards, in which case our estimates would identify a lower-bound estimate. The opposite is true for effects on health at birth outcomes, which are positively affected by higher birth orders, although these effects dissipate by age 7 (Pruckner et al., 2021; Brenøe and Molitor, 2018). Nevertheless, we hope to absorb these effects by including birth order dummies in our models.

While much of the negative selection into early motherhood is accounted for by our MFE models, these models would be able to recover the causal effect of early motherhood only in the absence of systematic sibling-specific unobserved heterogeneity. In this respect, three concerns are worth mentioning. First, differences in maternal behavior by sibling, that correlate with early motherhood status but are not conceptually driven by maternal age, represent an identification threat. For instance, parents that observe early human capital differences across siblings by early motherhood status could engage in either compensating or reinforcing behavior *independently from maternal age*.^21^ Second, time-varying household-level covariates that are not related to the mother’s aging also represent a threat for identification. If, for instance, the household significantly improved its socioeconomic status between rounds independently from mother’s age, the younger sibling would then be exposed to a better environment at earlier ages than his/her sibling would. While the age fixed effects account for the overall socioeconomic trend, household-specific socioeconomic progress over time remains a concern. Third, sibling interaction might alter the human capital gap between them associated with maternal age differences.

To ease such concerns inherent to sibling-difference models, we carry out four additional exercises. First, we apply the Oster (2019) method to investigate the empirical relevance of child-specific unobservables for our estimates. The Oster method is a useful and particularly powerful tool in a setting of sibling-difference models, as recognized by Aizer et al. (forthcoming). The test draws on coefficient and *R*-squared movements to identify the delta statistic, which stands for the ratio of selection on unobservables to selection on observables which would make the coefficient of interest equal to zero. Oster (2019) generally indicates values larger than 1 as evidence for the presence of robust effects. Such values would indicate that for the effect to be zero, the role of what is unobserved in a specific dataset would have to be larger than the role of observables in explaining selection. In our case, such a delta value would imply that child-specific factors within a household would have to play a bigger role than age, gender, birth order effects, and all observed and unobserved household and mother-level factors shared by siblings for the coefficient of adolescent mothers to be zero. Values significantly below a unit on the other hand would represent a threat to our estimates.^22^

Second, we carry out falsification tests to investigate whether we obtain similar results from equivalent sibling comparisons but with higher maternal age cut-offs located in adulthood Figure A3A4. If inherent aspects of the sibling comparison and not the early motherhood effect are driving our results, and then we should observe negative estimates for higher maternal age cut-offs located in adulthood. By contrast, the absence of such estimates would reinforce our early motherhood narrative, as we have no reason to assume a shift in the influence of these inherent factors by early motherhood status yet at the same time unrelated to early motherhood.

Third, we test whether older siblings are generally systematically (dis)favored. As the child born to the adolescent mother is coincidentally the older sibling for a subsample of sibling pairs, our estimate of interest could be driven by an older sibling effect. Hence, a negative and statistically significant coefficient for the older sibling dummy would suggest a generalized detrimental effect of being the older sibling, posing an identification threat to our analysis.

Fourth, household-specific socioeconomic progress over time could be systematically favoring the development of the later born child, potentially confounding our estimates. We therefore control in a robustness test for the exposure to household shocks, a household wealth index based on very rich information, and household consumption during the early childhood period of each child.

Finally, although our MFE model attempts to isolate the net effect of maternal age, it shares an important limitation with other studies exploring these effects on children and adolescents. These results are likely affected by selection biases related to mortality rates among young mothers and their offspring, as health and cognition data on children who have died are naturally missing.^23^ This is an important consideration given that the leading cause of death for teenage girls is pregnancy (WHO 2016). Moreover, the fetal, neonatal, and infant mortality are likely not uniformly distributed. Children born to teen mothers are at higher risk of being born underweight and premature and ultimately face a higher risk of infant mortality (Neal et al. 2018; Conde-Agudelo et al. 2005). This survival selection would bias our estimates towards zero. 24

## Results

### Adolescent motherhood and offspring outcomes

The OLS and MFE estimated effects of being born to an adolescent mother on offspring outcomes are shown in Table 3. The first two columns report estimates for height-for-age z-scores (HAZ) as a dependent variable, while the last two show estimates for math IRT scores.

**Table 3:** Regression results: adolescent motherhood and offspring outcomes

	HAZ		Math	
	(1)	(2)	(3)	(4)
	OLS	MFE	OLS	MFE
Adolescent mother	−0.22***	−0.23***	−13.73**	−1.57
	(0.06)	(0.08)	(5.93)	(6.01)
Delta (*β* = 0)		116		
*R*-squared	0.13	0.59	0.21	0.68
Observations	8630	8698	5822	5872

Clustered standard errors at mother level are in parentheses. The sample includes sibling pairs for rounds 3, 4, and 5 for HAZ regressions and rounds 4 and 5 for math regressions. Adolescent mother refers to children born to mothers under 18 years of age. The reference category is the maternal age group of mothers 18 years old and older at the time of childbirth. The dependent variables are HAZ (z-scores) and math (IRT scores). All regressions control for dummies for age, gender, birth order, and round. The OLS regressions include ethnicity, mother’s height, and rural/urban status in round 1. The MFE regressions control for mother fixed effects. The statistic delta is obtained through the STATA command psacalc (Oster 2019)

* *P* < 0.1; ** *P* < 0.05; *** *P* < 0.01

The OLS regression in column 1 suggests that being born to an adolescent mother is associated with 0.22 lower HAZ on average, compared to children born to adult mothers, with the estimated coefficient being significant at the 1% level). Notably, controlling for mother fixed effects barely alters these results.^25^ The point estimate remains highly significant and amounts to −0.23. For the average offspring age in our sample of 11.2 years; this implies a penalty of 1.57 cm for boys and 1.54 cm for girls, according to WHO Child Growth Standards (2007).^26^ While relatively moderate, such differences might be quite relevant for the development of vulnerable children.

Given the significant jump in the explanatory power of the model caused by the inclusion of mother fixed effects, the stability of the coefficient of interest is remarkable. We perform the Oster method to derive formal implications of these movements and report the delta statistic at the bottom of Table 3.^27^ The test results suggest that for the true effect of being born to an adolescent mother to be zero, selection on child unobserved heterogeneity would have to be significantly larger than selection on controlled factors, which in this case include age, gender, birth order, and all observed and unobserved mother and household characteristics shared by siblings. As the latter factors are established key determinants of child anthropometrics and early motherhood, we argue that such an assertion is rather implausible. Hence, this result strongly supports the conclusion that adolescent motherhood is detrimental to offspring HAZ.

To shed light on our interpretation of the negative HAZ results, we plot coefficients from the falsification tests in Fig. A3 in the Appendix. To produce these estimates, we use the same specification as in Table 3, but with maternal age cut-offs at 21 years and above. If we were to find similar results to those reported in Table 3, this would raise concerns about the estimated adolescent motherhood effect. As shown in Fig. A3, the falsification tests fail to reproduce our main results for HAZ. We do not observe a single negative and statistically significant coefficient for these alternative maternal age cut-offs. Hence, this suggests that inherent factors of the sibling comparison that are unrelated to early maternal age are not driving our results, reinforcing the early motherhood interpretation of our estimates. Note that in particular given that results suggest that sibling-specific unobserved heterogeneity does not play a role in our estimates, the differences between the estimates shown in this figure and the ones presented in our main results can be also interpreted as evidence of a non-linear effect of maternal ages.

As being born to an early mother coincides with being the older sibling for a subsample of sibling pairs, we further investigate whether older siblings are generally systematically disfavored.^28^ In Table A4 in the Appendix, the point estimates for being the older sibling are positive and statistically insignificant in columns 1 and 2. This suggests that there is no generalized pervasive effect for older siblings, which further supports the maternal age narrative. Furthermore, as shown in columns 3 to 7, our estimate is robust against controlling for time-varying family-level covariates that are contemporaneous to the early childhood of each sibling, including the number of shocks, the family wealth index and (real per capita) total expenditure.^29^ Point estimates are barely affected, which suggests that household-specific socioeconomic progress over time is unlikely to be driving our results.

Turning to the math results, columns 3 and 4 of Table 3 show that the evidence for a detrimental effect of being born to an adolescent mother is weaker for cognition outcomes, as measured by math IRT scores. The OLS estimates suggest that children born to adolescent mothers perform worse in the math test by 0.12 SD on average. However, this effect is not robust to the inclusion of mother fixed effects, suggesting that the detrimental effect is the result of unobserved selection into early motherhood rather than signaling a negative effect of early maternal age on children’s cognition.^30^

Given well-documented linkages between health and cognition, a reasonable prior would be to observe similar tendencies for both outcomes (Lo Bue 2019; Bharadwaj et al. 2018; Sánchez 2017; Sudfeld et al. 2015; Spears 2012; Grantham-McGregor et al. 2007). However, we find evidence for a detrimental effect for HAZ but no effect for math. A potential explanation for this combination of results relates to measurement errors. If math skills are measured with more classical error than HAZ, attenuation bias would make it harder to detect significant estimates in math regressions. Andersen et al. (2015) speculate on the (in)sensitivity of cognitive test scores to explain a similar combination of results. In addition, if health aspects are closer in the causal chain of interest than cognition aspects, i.e., the impact of early motherhood is more directly affecting child’s health than skills development, systematic relationships would be easier to detect in the case of health outcomes.

### Dynamics over time

We now attempt to shed light on the dynamics of the effect of adolescent motherhood over time, taking advantage of having repeated measures of the same developmental indicators.

There is extensive evidence on the importance of early childhood circumstances for education, employment, and health outcomes in adulthood. The existing evidence supports the widespread perception that it is cost-effective to focus on the very young ages. However, there is little evidence on the efficacy of programs targeting different age groups and on the impact of early childhood circumstances on middle childhood and adolescence outcomes, which Almond et al. (2018) refer to as the “missing middle.”

The Young Lives data offers the opportunity to shed light on the dynamics of the effect of early life circumstances over time. This paper contributes to filling in the gap in the literature investigating the effect of adolescent motherhood over time, taking advantage of having repeated measures of the same developmental indicators at different developmental stages. Considering the age range of our sample and the scarce evidence on the effects of early circumstances on development outcomes during the transition from middle childhood to adolescence, estimating the trajectories of these effects is particularly informative (Almond et al. 2018). In contrast to studies focusing on a single cross-section, this allows us to get a wider perspective on the relationship at hand. It tells us in which of the childhood stages covered are effects observed and whether these effects tend to accumulate or diminish over time. For instance, there might be early factors that affect children during middle childhood but not in adolescence due to catching-up dynamics (see Jones et al. (2018) for catch-up estimates using Young Lives data). Conversely, associations that remain latent through middle childhood and become apparent only in early adolescence due to cumulative processes in child development are also possible (Levine et al. 2007; Cunha et al. 2006). The panel nature of our data and our study design let us identify these trajectories.

We present these results in Table 4, which adds a set of interactions between the dummy for being born to an adolescent mother and data rounds. The round used as base category is the earliest available for each outcome variable. Hence, the coefficient for adolescent mother refers to round 3 for HAZ regressions and to round 4 for math regressions. Coefficients of the interactions with the round dummies indicate the change of this effect over time, whereas the sum of the coefficient for adolescent mother and the interaction coefficient gives the early motherhood effect at each respective round. Corresponding *P*-values from *t*-tests are reported at the bottom of the table. We also interact the vector of controls with the round dummies, which allows for their influence to vary over time.

**Table 4:** Regression results: adolescent motherhood and offspring outcomes over time

	HAZ		Math	
	(1)	(2)	(3)	(4)
	OLS	MFE	OLS	MFE
Adolescent mother	−0.30***	−0.33***	−9.20	5.76
	(0.07)	(0.08)	(6.64)	(6.38)
Adolescent mother × round 4	0.13**	0.14***		
	(0.05)	(0.05)		
Adolescent mother × round 5	0.15**	0.14**	-6.92	-12.97*
	(0.07)	(0.06)	(7.05)	(6.65)
*P*-value (*β* = 0, round 4)	0.01	0.02		
*P*-value (*β* = 0, round 5)	0.02	0.03	0.02	0.33
*R*-squared	0.15	0.59	0.22	0.69
Observations	8590	8698	5794	5872

Clustered standard errors at mother level are in parentheses. The sample includes sibling pairs for rounds 3, 4, and 5 for HAZ regressions and for rounds 4 and 5 for math regressions. Hence, rounds 3 and 4 are the reference rounds for HAZ and math regressions, respectively. Adolescent mother refers to children born to mothers under 18 years of age. The reference category is the maternal age group of mothers 18 years old and older at the time of childbirth. The dependent variables are HAZ (z-scores) and math (IRT scores). All regressions control for dummies for age, gender, birth order, and round. The OLS regressions include ethnicity, mother’s height, and rural/urban status in round 1. The MFE regressions control for mother fixed effects. All regressions include round interactions with controls

* *P* < 0.1; ** *P* < 0.05; *** *P* < 0.01

OLS and MFE of the HAZ regressions in Table 4 show very similar results. Both indicate that the detrimental effect of adolescent motherhood is largest in the earliest round. In the MFE model, the penalty associated with being born to an adolescent mother is of 0.33 HAZ and significant at the 1% level in round 3, when children are on average 8 years old. Interestingly, the magnitude of the effect decreases over time and remains significant at the 5% level in both subsequent rounds, suggesting that a partial catch-up has taken place during the transition between childhood and adolescence.^31^ In round 5, when children are on average 15 years old, the point estimate amounts to 0.19, which implies a penalty of 1.48 cm and 1.31 cm for boys and girls, respectively.^32^ Given that height growth is minimal after this age (WHO 2007), this estimate implies that the estimated negative association is highly likely permanent and could therefore reverberate to labor productivity effects later in life (LaFave and Thomas 2017).

Turning to the math results, OLS estimates suggest that the effect surges at a later stage, as the negative relationship is statistically significant in round 5 but not in round 4. However, the association is weaker when accounting for MFE. The MFE point estimate is not statistically significant in any of the rounds. The estimated coefficient for round 4 is positive and turns negative in round 5. While it remains insignificant as indicated by its *P*-value, the interaction itself shows a downward trajectory that is statistically significant at the 10% level. Note that the two outcomes of interest show different trajectories, pointing to the coexistence of physiological catch-up growth and self-productivity dynamics of cognition.

### Young mothers, very young mothers and gender

So far, we have compared children born to adolescent mothers with those born to adult mothers, ignoring that there might be important differences within the adolescent mothers’ group. Since we find that early maternal ages are detrimental to offspring development, it is worth investigating whether the effect of early motherhood is stronger for those children born to the youngest mothers among adolescent mothers. To explore this, we distinguish between children born to *very young* mothers (< 16 years old) and to *young* mothers (16–17 years old), previously combined into the adolescent mothers’ group. Moreover, we investigate heterogeneous effects by gender. Human capital investments in Indian children have been shown to be gender-skewed (Barcellos et al. 2014). More closely related to our exercise, previous research suggests that Indian households facing adverse circumstances favor sons over daughters (Asfaw et al. 2010; Rose 1999; Berhman 1988).

The results for HAZ and for math are presented in panels A and B of Table 5, respectively. The table shows OLS and MFE estimates, but for the sake of simplicity we will focus on the MFE estimates only. The first two columns report estimates that pool all data rounds. The third and fourth columns show results with round interactions to identify dynamics over time. The last two columns report results with gender interactions in order to explore heterogeneous effects by gender. The *P*-values from *t*-tests of the overall effects in each round and for each gender are reported at the bottom of each panel.

**Table 5:** Regression results: young, very young mothers and gender

	(1)	(2)	(3)	(4)	(5)	(6)
	OLS	MFE	OLS	MFE	OLS	MFE
*Panel A: HAZ*						
Very young mother	−0.32***	−0.37**	−0.45***	−0.54***	−0.53***	−0.65***
	(0.11)	(0.17)	(0.15)	(0.18)	(0.16)	(0.19)
Very young mother × round 4			0.23*	0.21*		
			(0.13)	(0.12)		
Very young mother × round 5			0.22	0.24		
			(0.16)	(0.15)		
Very young mother × boy					0.43**	0.60**
					(0.21)	(0.29)
Young mother	−0.20***	−0.21***	−0.29***	−0.30***	−0.20**	−0.20*
	(0.06)	(0.08)	(0.07)	(0.08)	(0.08)	(0.12)
Young mother × round 4			0.12**	0.13**		
			(0.06)	(0.05)		
Young mother × round 5			0.15**	0.12*		
			(0.07)	(0.06)		
Young mother × boy					0.01	−0.01
					(0.11)	(0.15)
Delta (*β* = 0, very young mother)		1.56				
Delta (*β* = 0, young mother)		1.60				
*P*-value (*β* = 0, very young mother, round 4)			0.07	0.06		
*P*-value (*β* = 0, very young mother, round 5)			0.09	0.14		
*P*-value (*β* = 0, young mother round 4)			0.01	0.04		
*P*-value (*β* = 0, young mother round 5)			0.05	0.04		
*P*-value (*β* = 0, very young mother, boy)					0.44	0.86
*P*-value (*β* = 0, young mother, boy)					0.02	0.03
*R*-squared	0.13	0.59	0.14	0.59	0.13	0.59
Observations	8630	8698	8630	8698	8630	8698
*Panel B: math*						
Very young mother	−43.71***	−25.42*	−37.40**	−11.77	−49.93***	−36.07**
	(14.14)	(13.33)	(14.71)	(13.92)	(16.23)	(15.64)
Very young mother × round 5			−10.45	−22.16		
			(15.55)	(15.03)		
Very young mother × boy					21.61	15.80
					(22.86)	(23.98)
Young mother	−7.41	1.58	−3.76	7.64	−6.50	8.43
	(5.89)	(6.02)	(6.69)	(6.51)	(8.15)	(8.81)
Young mother × round 5			−6.13	−11.23		
			(7.28)	(6.95)		
Young mother × boy					−2.89	−12.21
					(10.70)	(11.24)
Delta (*β* = 0, very young mother)		2.79				
*P*-value (*β* = 0, very young mother round 5)			0.01	0.05		
*P*-value (*β* = 0, young mother round 5)			0.17	0.63		
*P*-value (*β* = 0, very young mother, boy)					0.10	0.32
*P*-value (*β* = 0, young mother, boy)					0.20	0.62
*R*-squared	0.21	0.68	0.22	0.69	0.38	0.72
Observations	5822	5872	5822	5872	6977	6945

Clustered standard errors at mother level in parentheses. Sample includes sibling pairs for rounds 3, 4, and 5 with round 3 as the reference round in panel A and sibling pairs for rounds 4 and 5 with round 4 as the reference round in panel B. Very young mothers and young mothers refer to children born to mothers under 16 years of age and 16–17 years old, respectively. The reference category is the maternal age group of mothers 18 years old and older at childbirth. The dependent variable is height for age (z-scores) in panel A and math (IRT scores) in panel B. All regressions control for dummies for age, gender, birth order, and round. The OLS regressions include ethnicity, mother’s height and the rural/urban status in round 1. The MFE regressions control for mother fixed effects. Columns 3 and 4 include round interactions with controls, whereas columns 5 and 6 include gender interactions with controls

* *P* < 0.1; ** *P* < 0.05; *** *P* < 0.01

Overall, there are three main messages from the HAZ analysis shown in panel A. First, children born to young and very young mothers tend to have lower HAZ than children born to adult mothers. The statistical significance of these associations varies depending on the round. Second, point estimates are lower for very young mothers, in line with our hypothesis. Third, effects generally weaken over time.

Estimates reported in column 2 of panel A indicate that children born to young and very young mothers have 0.21 and 0.37 lower HAZ than children born to adult mothers. Both coefficients are statistically significant. Moreover, they both show delta statistics significantly higher than unity, supporting the conclusion that adolescent motherhood is detrimental to offspring HAZ. While the point estimate for very young mothers is lower than the coefficient for young mothers, they are not statistically different from each other.^33^

When we look at the dynamics over time in column 4 of panel A, we observe the same pattern across maternal age groups. The strongest effects are observed in the earliest round and the magnitude of the effects decrease over time. Remarkably, the estimated HAZ penalty for children born to very young mothers in round 3 is approximately 1.8 times larger than the penalty experienced by children born to young mothers (0.54 versus 0.30, respectively), a ratio that slightly increases in round 4 and slightly decreases in round 5. These differences across early maternal age groups are, however, not statistically significant, as indicated by the *P*-values in Table A4 in the Appendix. All coefficients in column 4 remain significant at the 10% level in rounds 4 and 5, with the exception of the coefficient for very young mothers in round 5. Moreover, although point estimates for very young mothers are always larger in magnitude, estimation for this variable is usually less precise.

In column 6 of panel A, we report MFE results of a model that adds double interactions between the maternal age groups and gender. For children born to very young mothers, girls are substantially worse off. They show HAZ values that are 0.65 lower than their counterparts born to adult mothers, while coefficients for boys are negative but statistically insignificant. Moreover, negative effects for girls born to young mothers are statistically significant in rounds 3 and 5, while for boys they are significant in rounds 3 and 4, but turn insignificant in round 5. The differences in point estimates between girls born to very young mothers and those born to young mothers are statistically significant.^34^

The gender-skewed effects observed among children born to very young mothers are in line with the literature documenting parental responses favoring sons to adverse circumstances in India (Asfaw et al. 2010; Rose 1999; Behrman 1988) and Sub-Saharan Africa (Delprato et al. 2017). However, given the limited power we face for this gender analysis, some caution is suggested in interpreting the statistically insignificant results for boys born to very young mothers as evidence for null effects.

We now turn to the MFE results for math, reported in panel B. Overall, we identify two main messages. First, children born to very young mothers tend to perform worse in the math test than children born to adult mothers.^35^ This is not the case for the offspring of young mothers. Second, negative effects associated to very young mothers surge during early adolescence.

Interpreting these results in more detail, estimates in column 2 show that being born to a very young mother is associated with a decrease in math scores of 0.23 *SD* significant at the 10% level, compared to children born to adult mothers. Importantly, the delta statistic for the very young mother coefficient is substantially higher than 1. Looking at the dynamics over time in column 4, we observe that the effect turns statistically significant in round 5, when children born to very young mothers perform 0.31 *SD* worse.^36^ This pattern is consistent with the notion of skills self-productivity put forward by Cunha et al. (2006). In this view, a child slightly behind in his cognitive development is less productive at acquiring new stocks of cognitive skills, and might fall further behind as he or she progresses.

In column 6 of panel B, we explore heterogeneous effects by gender. Similar to the results for HAZ, girls of very young mothers do worse than boys in the math test. The magnitude of the statistically significant coefficient is now 0.33 SD. We again abstain from interpreting the insignificant effects for boys of very young mothers as null effects due to precision issues.

### Transmission channels

In this section, we explore some candidates possibly explaining the estimated relationship between early maternal age and offspring health and cognition. Building on human capital theoretical frameworks such as those in Attanasio (2015), Cunha et al. (2006), and Todd and Wolpin (2003), we hypothesize maternal age to enter the child’s human capital production function via two main pathways: the “behavioral channel” and the “biological channel.”

In relation to the behavioral channel and adopting Attanasio’s (2015) terminology, child outcomes depend on “parental investments” and “parental background” (i.e., parental characteristics), conditioned on child’s initial conditions and shocks occurring over time. Parental investments in human capital are themselves a function of parental characteristics, including maternal age, and observables and unobservable factors related to it, such as education and socioeconomic background, preferences, expectations, and psychological maturity. These factors are likely to affect mothers’ behaviors and practices, particularly in regard to prenatal care, childrearing practices and, more broadly, decisions around investments in child’s human capital.^37^ We follow the literature on intra-household resource allocation highlighting the role of mothers in human capital investments for their children and explore to what extent being an adolescent mother might imply having little knowledge and/or low bargaining power within the household, resulting in limited investments in children’s human capital (Doss 2013).

For the biological channel, we hypothesize that adolescent mothers are biologically immature for childbearing, which might negatively affect the initial human capital endowment of the child. Using Attanasio’s (2015) terminology, these disadvantaged initial conditions of biological nature would then negatively affect children’s subsequent human capital outcomes. Indeed, children born to young mothers face higher risks of poor neonatal outcomes such as preterm birth and low birthweight, among others (Neal et al. 2018; Fall et al. 2015; Gibbs et al. 2012; Neal et al. 2012; Conde-Agudelo et al. 2005). In turn, such poor neonatal outcomes have been associated with negative impacts on offspring anthropometrics, schooling and adult earnings (McGovern 2018; Black et al. 2007; Behrman and Rosenzweig 2004).

In this section, we investigate channels that are either biological or behavioral in nature. We employ regression analysis with the hypothesized channels for child’s health and cognition as dependent variables in order to investigate whether maternal age groups are systematically related to them. Our ability to explore these channels is limited by the quantity and quality of the information available either for the index children only or for the sibling pairs. In the first case we can only report OLS results. When data are available on the sibling pairs, we report both OLS and MFE estimates. While the results presented here are suggestive and hence should be cautiously interpreted and are not comprehensive in exploring the pathways through which early motherhood affects child’s human capital, they provide additional instructive insights.

To shed light on the mechanisms explaining the effect of maternal age on HAZ, we look at the variables of birthweight and dietary diversity.^38^ For dietary diversity, we follow the guidelines of Bilinsky and Swindale (2006) to construct the individual dietary diversity score, a measure of nutritional quality that reflects macro and micronutrient adequacies for children (FANTA 2006; Mirmiran et al. 2004). The 0–7 score counts the number of nutritionally meaningful food groups consumed in the previous 24 h by the child.^39^ We hypothesize that mothers’ knowledge of nutrition and cooking practices increases with age, as well as their bargaining power over the purchase and consumption of more adequate food items in the household. If this were the case, children born to adolescent mothers would systematically achieve lower dietary diversity scores, which in turn would affect their height-for-age (Mallard et al. 2014).

In Table 6, we report the results for both birthweight and dietary diversity. OLS estimates for birthweight show that being born to a very young mother is associated with a decrease in birthweight by 176 g, at the 5% level of significance. Controlling for mother fixed effects results in a stronger and statistically significant effect of 307 g, which is considerable, given that the average birthweight in the sample is of 2770 g^40^ That is, for the average child, the effect would imply falling below 2500 g into a low birthweight category as defined by the WHO (2004). Furthermore, the delta statistic is significantly higher than the threshold level of 1, supporting the causal interpretation of our estimate. However, it is worth acknowledging that birthweight data are missing for more than half of our sample, an important limitation to be considered in the overall assessment of these results.^41^

**Table 6:** Regression results: exploring the transmission channels for health

	Birthweight		Dietary diversity
	(1)	(2)	(3)
	OLS	MFE	OLS
Very young mother	−176.21**	−306.59**	−0.39**
	(86.17)	(152.88)	(0.18)
Very young mother × round 4			0.31
			(0.22)
Very young mother × round 5			0.49*
			(0.30)
Young mother	12.26	33.26	−0.06
	(52.02)	(70.45)	(0.07)
Young mother × round 4			0.06
			(0.11)
Young mother × round 5			0.03
			(0.11)
Sample	Sibling pairs	Sibling pairs	Index child
Delta (*β* = 0, very young mother)		6.89	
*P*-value (*β* = 0, very young mother, round 4)			0.70
*P*-value (*β* = 0, very young mother, round 5)			0.67
*P*-value (*β* = 0, young mother, round 4)			0.96
*P*-value (*β* = 0, young mother, round 5)			0.76
*R*-squared	0.03	0.60	0.05
Observations	1421	1428	5625

Clustered standard errors at mother level are in parentheses. The sample consists of the sibling pairs for birthweight regressions and of index children in rounds 3, 4, and 5 for the dietary score regression, with round 3 as reference round. Very young mothers and young mothers refer to children born to mothers under 16 years old and 16–17 years old, respectively. The reference category is the maternal age group of mothers 18 years old and older at the time of childbirth. The dependent variables are birthweight (grams) and individual dietary diversity score (0–7 range). OLS regressions control for gender, birth order, ethnicity, rural/urban status in round 1, and mother’s height. The dietary score regression controls in addition for age and round dummies. The MFE regression controls for mother fixed effects. The statistic delta is obtained through the STATA command *psacalc* (Oster 2019)

* *P* < 0.1; ** *P* < 0.05; *** *P* < 0.01

OLS estimates for dietary diversity shows that at the age of 8, children born to very young mothers achieve lower dietary diversity scores than those born to adult mothers. They consume 0.39 (0.46 *SD*) fewer food groups, which constitutes a modest but non-negligible difference considering that on average children in round 3 consume 4.35 food groups daily. Interestingly, the correlation weakens over time, suggesting that as very young mothers’ age, the diet quality of their children improves. Moreover, these results emulate the trajectory of our results for HAZ, as the effect of maternal age decreases as the child grows up. Overall, we interpret these estimates as suggestive evidence for birthweight and dietary diversity as a mediation channel for children born to very young mothers.

We now look at the transmission channels for child’s cognition. We focus on the children born to very young mothers only, as these children are the ones found to perform worse in the math test, compared to children born to adult mothers. We investigate whether slow school grade progression (or being overage-for-grade), education expenditure, and maternal involvement in child’s education behave as mediating channels.

Overage-for-grade is a dummy that indicates whether the child is overaged for the school grade she is enrolled in at the start of the school year, taking into account the official entrance age for each grade in the states of Andhra Pradesh and Telangana. The rationale is that if children born to adolescent mothers experience lower and inefficient investments in human capital, they would tend to fall behind in school, increasing their likelihood of being overaged. This in turn would flatten their learning curves, creating a vicious cycle in which overage would be both a cause and a consequence of poor cognition (see UNESCO (2012) and Alexander et al. (2003) for suggestive evidence and conceptual discussions).

Education expenditure and maternal involvement in child’s education are our most direct proxies for parental investments in education. The former is defined as the share of total household expenditure per capita in real terms assigned to educational fees, including both school fees and private tuition fees. The latter is a dummy variable indicating whether the mother knows the name of the child’s teacher. Presumably, this variable correlates with mother-teacher meetings, which reflect the value that mothers place on their child’s education and has been linked to significant improvements of learning outcomes (Islam 2019).

In Table 7, OLS estimates suggest that being born to a very young mother is associated with an increase in the likelihood of being overaged by 11 percentage points at the 10% level in the earliest round. The association turns significant at the 5% level in round 4 and turns statistically insignificant in round 5. In the MFE results, the association is significant at the 5% level only in round 4. At this round, the likelihood of being overage-for-grade increases by 18 percentage points for children born to very young mothers. However, the coefficient is not statistically significant in round 5, such that overage is unlikely to be a transmission channel.

**Table 7:** Regression results: exploring the transmission channels for cognition

	Over age for grade		Education expenditure	Teacher’s name
	(1) OLS	(2) MFE	(3) OLS	(4) OLS
Very young mother	0.11*	0.12	−0.01	0.01
	(0.06)	(0.08)	(0.02)	(0.10)
Very young mother × round 4	0.03	0.06	0.00	−0.19
	(0.06)	(0.05)	(0.05)	(0.15)
Very young mother × round 5	−0.01	0.03	−0.02	
	(0.11)	(0.10)	(0.06)	
Sample	Sibling pairs	Sibling pairs	Index child	Index child
*P*-value (*β* = 0, very young mother, round 4)	0.03	0.04	0.85	0.05
*P*-value (*β* = 0, very young mother, round 5)	0.29	0.19	0.60	
*R*-squared	0.09	0.56	0.15	0.14
Observations	6000	6046	5350	3718

Clustered standard errors at mother level are in parentheses. The sample consists of the sibling pairs for overage regressions and of index children in rounds 3, 4, and 5 for the remaining columns. Round 3 is the reference category throughout. Very young mother refers to children born to mothers under 16 years old. The reference category is the maternal age group of mothers 18 years old and older at the time of childbirth. The dependent variables are a dummy for over age for grade, the share of real education expenditure on the index child in total expenditure of the household and a dummy indicating whether the mother knows the name of the child’s teacher. All regressions include an indicator for being born to young mothers and its interaction with the rounds. OLS regressions control for dummies for age, gender, birth order, ethnicity, rural/urban status in round 1, and in addition for mother’s height. The education expenditure regression includes total expenditure per capita in real terms. The MFE regression controls for dummies for age, gender, birth order, rounds, and in addition for mother fixed effects

* *P* < 0.1; ** *P* < 0.05; *** *P* < 0.01

Turning to our proxies for parental investments, we do not find statistically significant effects for educational expenditures. However, we do find suggestive evidence for teacher’s name estimates. The coefficient for very young mothers is small and insignificant in round 3. However, the point estimate turns large in magnitude and significant at the 10% level when children are 12 years old on average (round 4). At this round, very young mothers at birth are 18 percentage points less likely to know the name of the child’s teacher. This suggests that the gap in these types of investments between adult mothers and very young mothers surges during late-middle childhood.

## Conclusion

This paper investigates the effect of early maternal age on offspring human capital development during childhood and early adolescence. To circumvent identification issues related to mother unobserved heterogeneity, we estimate the effect of being born to an adolescent mother by comparing the offspring outcomes of children born to the same mother, thereby exploiting within-mother variation of maternal age at birth. We further ease remaining concerns on child-specific unobserved heterogeneity within households and net of age, gender and birth order effects with the Oster method, falsification tests, and further specifications exploring the role of early childhood conditions and the older sibling effect.

Our health analysis suggests that early maternal age is causally and negatively associated with offspring HAZ. In the earliest data round, when children are on average 8 years old, being born to an adolescent mother is associated with 0.33 lower HAZ, compared to children born to adult mothers. This detrimental effect weakens over time but remains statistically significant until early adolescence, suggesting both a partial catch-up and permanent effects. Moreover, point estimates for children born to very young mothers are larger than the effects for children born to young mothers, particularly among girls.

In terms of cognition, while we find no effect associated with being born to adolescent mothers, children born to very young mothers perform worse than children born to adult mothers (0.23 *SD*). However, our falsification tests and sample size considerations do not allow us to be as conclusive on the early motherhood interpretation of these estimates as for the case of HAZ regressions. Moreover, the math effect surges over time, amounting to 0.31 SD in round 5, when children are on average 15 years old. Similar to the HAZ results, girls born to very young mothers perform particularly worse in the math test.

Given that our estimates appear to be larger than those estimated for a developed country context (Aizer et al. forthcoming), the role of institutional and family safety nets in compensating for the detrimental effects of early motherhood in both LMICs and high-income countries should be explored in future research.

We further investigate behavioral and biological transmission channels. Although limited in its scope, our analysis provides suggestive evidence on the role of birthweight, food diversity, and maternal involvement in education as mediating factors behind the estimated detrimental effects. Further research should engage with a broader investigation of potential transmission channels and with the relative importance of behavioral vis-à-vis biological channels, which might have important policy implications.

Finally, we provide instrumental motivation to implement preventive measures that reduce early maternal age, complementing intrinsic concerns of early pregnancy related to human rights issues. In this vein, our analysis highlights the importance of law enforcement of the Indian Penal Code, which prohibits sexual intercourse for individuals under 18 years of age. A lack of law enforcement would contribute to the survival of adolescent motherhood and its pervasive intergenerational consequences. Policies that aim at effectively enforcing this sensitive law are therefore of relevance for all unborn generations yet to come. Finally, our results support restorative policy measures assisting early mothers and their offspring to lower the burden of early motherhood and foster the human capital of children.

## Supplementary Information

Below is the link to the electronic supplementary material.

Supplementary file1 (PDF 346 KB)

## Footnotes

1 The COVID-19 pandemic might increase the prevalence of this phenomenon via increased poverty, school closures keeping girls out of a protective environment, and the disruption of family planning services. According to the United Nations Population Fund, the pandemic could cause 13 million additional child marriages — a key driver of early motherhood (UNFPA 2020). In line with this rationale, spikes in adolescent pregnancy cases were observed during the Ebola outbreak in Sierra Leone (Elston et al. 2016).

2 Hence, while previous research has shown that early motherhood is correlated with poor offspring outcomes, there is a lack of consensus on the causality of this association. See Azevedo et al. (2012) for a review and Levine et al. (2007) and Aizer et al. (forthcoming) for examples of contrasting conclusions.

3 Such sibling-difference models are established empirical tools used in studies on human capital production (see for instance Kreiner and Sievertsen (2020); Autor et al. (2019, 2016); Helgertz and Nilsson (2019); Brenøe and Lundberg (2018); Black et al. (2016); Figlio et al. (2014); and on the literature on the consequences of maternal age on offspring outcomes using data from high-income countries such as Norway (Aizer et al. forthcoming), Sweden (Carslake et al. 2017), the UK (Francesconi 2008), and the US (Levine et al. 2007; López-Turley 2003; Rosenzweig and Wolpin 1995; Geronimus et al. 1994).

4 Such effects are considered as large ones by the education literature on LMICs (Evans and Yuan 2020; Muralidharan et al. 2019). However, we advise caution in the over-interpretation of our cognition estimates given the limited number of observations they draw upon, as well as in overemphasizing the comparison of these cognition effects with other studies, given the possibility of differing distributional properties of test scores (see Ost et al. 2017).

5 In particular given that these alternative specifications do not show a general pattern of negative effects, thereby reducing concerns about sibling-unobserved factors, the differences between these estimates and our main results can be understood as evidence of non-linear maternal age effects.

6 Two other studies look at the effect of marriage age instead and find that delaying marriage age is beneficial for offspring development (Chari et al. 2017; Delprato et al. 2017). While closely interrelated, marriage age and maternal age at birth are not equivalent. In a sample of Indian states, for instance, only 34% of married women aged 15–19 have given birth (IIPS 2017). One advantage of focusing on maternal ages is that, unlike marriage age, they are child-specific rather than family-specific, allowing us to exploit sibling-difference models that purge out family-level confounders.

7 The districts are Srikakulam and West Godavari in Coastal Andhra, Anantapur, and Kadapa in Rayalaseema, and Karimnagar, Mahbubnagar, and Hyderabad in Telangana.

8 In 746 cases out of 754 for which a younger sibling was not available, the index child was the youngest child in the household.

9 The index children with available sibling data are very similar to the overall sample of index children, based on household and offspring characteristics (see Table A3 in the Appendix).

10 The remaining 26 households are composed of same-aged siblings.

11 The number of sibling pairs across rounds is stable. See Table A1 in the Appendix for observations per round.

12 Child’s age is observed in months, whereas mother’s age is reported in years during 5 data collection rounds. As interview dates might capture the mother’s age at the beginning or end of a year cycle, slight differences in maternal ages might arise across rounds for the same child. We therefore calculate maternal age at birth by averaging the differences between mother’s age and child’s age across rounds. For more than 91% of the sample, there is no difference between the round-specific maternal age and the average maternal age across rounds. For almost 9% of the sample, the difference is of 1 year. By averaging across rounds, we therefore attempt to solve this minor issue for the latter subsample.

13 Mothers that are 18 years old or older at the time of birth are defined as adult mothers. The cut-off of 18 years old is based on the legal age of sexual consent, which is anchored in the Indian Penal Code, as well as on the age of legal independence of individuals in India. The 16-year-old cut-off is guided by the medical literature, which suggests that girls under the age of 16 are at higher risk of eclampsia, anemia, postpartum hemorrhage, obstetric fistula, obstructed labor due to underdeveloped pelvic bones, and worse neonatal outcomes than older adolescents (Criminal Law Act 2013; Neal et al. 2012).

14 An additional variable that could systematically vary by maternal age groups is marital status. However, in round 1 of data collection, when the index child was on average 1 year old, only eight mothers in total were identified as divorced, separated, single or widowed.

15 We follow WHO guidelines (2006) and set values out of the −6:6 range to missing due to their biological implausibility. We also correct for measurement error in height-for-age by dropping 34 observations that suggest an implausible decrease in absolute height over time. Note that this well-nourished population is drawn from 6 countries across the world, one of which is India (WHO, 2006).

16 See Fig. A1 in the Appendix for the means by rounds.

17 Attanasio (2015), extending the model by Cunha et al. (2006), is the only model that explicitly considers the health dimension as a separate element of human capital and is therefore our preferred theoretical reference.

18 A disadvantage of this procedure relates to the fact that by dropping between-mother variation, we cancel important channels through which early motherhood might affect offspring development, such as lower mother’s education due to pregnancy-induced school dropouts (e.g., Grépin and Bharadwaj 2015). OLS estimates remain robust to the inclusion of family size as an additional control; these results are available upon request.

19 The ability of our age fixed effects to absorb cohort effects is further supported by the overlapping support in age distributions of children born to adolescent mothers and to adult mothers, as shown in Fig. A2. Furthermore, our results and conclusions are robust to clustering the standard errors at the child-level.

20 Girls reach most of their adult height by the time of puberty, such that it is reasonable to assume that mother’s height is predetermined to the offspring’s birth (WHO 2007). Similarly, the rural/urban location of residence of the household in round 1 is in the vast majority of the cases the same at the time the mother conceived the index child.

21 The related literature has found empirical evidence for both reinforcing and compensating behavior. Note that the former would tend to overestimate the effect, while the latter would underestimate it (see Almond et al. (2018) for a review, and Fan and Porter (2020) and Bharadwaj et al. (2018) for examples of compensating behavior in health and cognitive skills).

22 Note that while we do not observe all time-invariant household and mother-level factors per se, we are able to account for them in the MFE models. Therefore, these factors act as observables in this exercise. By design, we are able to perform this test for non-interacted coefficients.

23 In India, the maternal mortality rate was estimated at 174 deaths per 100,000 live births in 2015 (WHO et al. 2015). Perinatal mortality amounted to 36 deaths per 1000 pregnancies in 2015–2016 (IIPS 2017).

24 An additional concern of family-fixed effects estimations is the exacerbation of attenuation bias stemming from classical measurement error in explanatory variables (Griliches 1979). However, we conjecture that the role of classical measurement error is rather limited in the measurement of adolescent motherhood status.

25 This can be the result of biases discussed in Section 3 canceling each other out. Alternatively, selection mainly occurring on observables might be responsible for these results. For this to be a possibility, OLS estimates should react to the inclusion of controls, which happens in our case (estimates available on request).

26 To put this in perspective, Aizer et al. (forthcoming) use sister fixed effects and Norwegian data to estimate a gain of 0.6 cm for boys born to non-teen mothers. Schroeder et al. (1995) report that in Guatemala, being randomly exposed to a protein-rich food supplement for 3 years starting from birth and on a twice-daily basis resulted in a positive treatment effect of 2.5 cm by the end of the exposure period, while the 0–12-year-old offspring of the treated children also benefited with average gains of 0.26 HAZ (Behrman et al. 2009). Miguel and Kremer (2014) find in their randomized control trial that after a year of deworming treatment for Kenyan pupils of grades 3–8, they gained 0.08 HAZ, compared to children with no treatment. However, Baird et al. (2016) find no effect on height 10 years later. Behrman and Hoddinott (2001) use child fixed effects to find that infant children participating in the Progresa program in Mexico, which combines conditional cash transfers, nutritional education, and micronutrient-fortified supplements, gained an additional 1 cm per treated year. Similarly, boys exposed to Juntos program, a cash transfer program in Peru conditional upon health care visits for more than 2 years, gained 0.43 HAZ at ages 7–8 years (Andersen et al. 2015).

27 For this exercise, we follow Oster’s guidelines (2019) and set *R*_max_ to 1.3 $\overset{\sim }{R}$.

28 Note that the older sibling variable indicates who of the two siblings in the pairs is older, whereas the birth order dummies indicate their birth order considering all siblings. Given that we also look at mothers with more than two children, these two variables are not collinear.

29 See Briones (2018) for a detailed description of shocks and the wealth index.

30 In Table A4 and in Fig. A4 in the Appendix, we show that our robustness checks do not change this result.

31 This is consistent with recent studies suggesting catch-up growth dynamics during childhood (Anand et al. 2018; Desmond and Casale 2017).

32 This effect is more than 2.4 times larger than those estimated for a developed country context such as Norway (Aizer et al. forthcoming). This differential could be for instance linked to a younger profile of adolescent mothers in LMICs or to compensation mechanisms available in developed countries, such as social safety nets.

33 *P*-values of these tests are available upon request.

34 *P*-values of these tests are available upon request.

35 However, the falsification tests for math shown in Fig. A4 in the Appendix provide mixed evidence on the early motherhood interpretation of this estimate. On the one hand, most of the coefficients from alternative maternal age cut-offs fail to reproduce a negative and statistically significant estimate, which speaks for the early motherhood narrative. On the other hand, the cut-off at 30 years produces such a coefficient. This illustrates that, while seldom, other factors unrelated to early maternal age can produce negative estimates. As this is not a general pattern, it could also speak for non-linear effects of maternal age shifting at the value of 30. Finally, the number of observations that contribute to our math estimates is limited raising caution about their conclusiveness.

36 Aizer et al. (forthcoming) use Norwegian data and sister fixed effects to estimate that being born to a 15–17-year-old mother is associated with a decrease of 0.18 *SD* in an IQ test. As an additional reference point, the effect size we estimate is considered as a large one in the education literature using experimental methods in LMICs (Evans and Yuan 2020; Muralidharan et al. 2019). However, we advise caution in overemphasizing the comparison of these effects. As the distribution of scores are not constant across studies, similar SD movements might stand for different cognition gains in absolute terms (see Ost et al. (2017) for an analysis of this issue).

37 Some of these factors, such as parental socioeconomic background, do not vary across siblings and hence speak only to OLS estimates. Others, such as psychological maturity, change over time and hence vary across siblings’ exposure to parenting. For instance, Icenogle et al. (2019) suggests that psychosocial maturity continuously increases with age, using data from individuals between 10 and 30 years in India and other 10 countries. This type of variation speaks to MFE estimates.

38 Although we portray birthweight as a potential transmission channel for health outcomes, it also embodies a potential mechanism for cognition (Figlio et al. 2014).

39 See Table A2 in the Appendix for a detailed description of this and other variables.

40 Note that controlling for birth order fixed effects is essential to avoid a downward bias here, as previous studies have documented a positive effect of higher birth order on health at birth (Brenøe and Molitor, 2018).

41 We also show in Table A5 in the Appendix that birthweight data is not missing randomly, as children born to very young mothers are more likely to have missing birthweight data.
