# Supplementary material for: Children having children: early motherhood and offspring human capital in India
Source: J Popul Econ. 2023 Mar 28;36(3):1573–606. doi: 10.1007/s00148-023-00946-0 (PMC10043537; doi:10.1007/s00148-023-00946-0)
Supplement: Supplementary file 1 — Supplementary file1 (PDF 346 KB) [file 148_2023_946_MOESM1_ESM.pdf]

Online Appendix for paper
“Children Having Children:
Early Motherhood and Offspring Human Capital in India”

Figure A1. Mean of HAZ and math by round and maternal age group

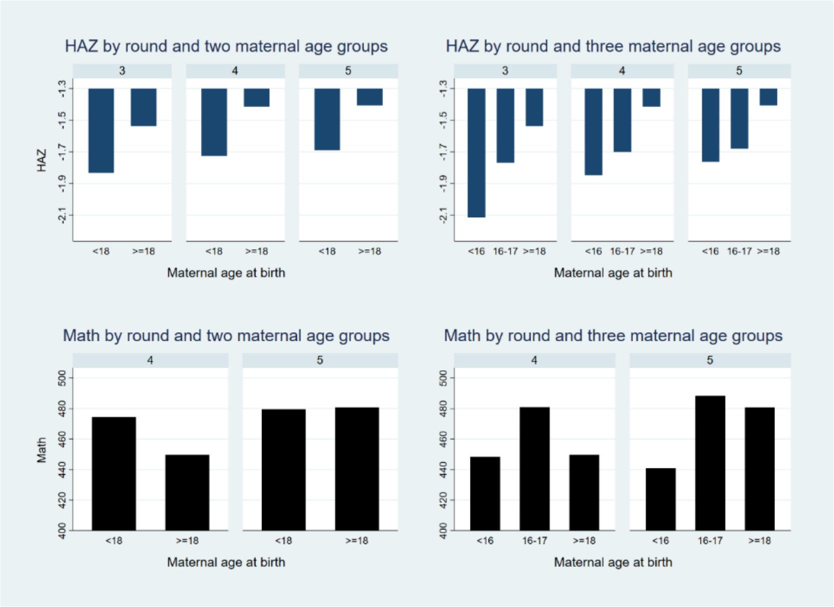

Notes: Statistics refer to child-round-level observations with data on age, gender, birth order, maternal age and the respective outcome variable for the sibling pairs participating in Rounds 3-5. Maternal age groups are shown on the x-axis. HAZ is height-for-age in z-scores, while math consists of IRT scores.

Table A1. Age of index children and siblings by round

		Index children			Siblings		
		Mean	SD	N	Mean	SD	N
Child age	Round 3 (2009)	8.0	0.3	1,535	7.9	3.2	1,535
	Round 4 (2013)	12.0	0.3	1,607	11.7	3.5	1,607
	Round 5 (2016)	15.0	0.3	1,462	14.5	3.6	1,462

Notes: Sample per round is restricted to households with sibling pairs information on age, gender, birth order and height-for-age or math scores.

Table A2. Detailed description of variables

Variable	Description
Maternal age variables	
Maternal age at birth	Mother's age at the time of childbirth. This variable is constructed by taking the average of the differences between the child's age (reported in months) and mother's age (reported in years) across data rounds.
Adolescent mother	Binary indicator taking the value of 1 for children born to mothers under the age of 18 at the time of childbirth and 0 otherwise.
Young mother	Binary indicator taking the value of 1 for children born to mothers that were 16-17 years old at the time of childbirth and 0 otherwise.
Very young mother	Binary indicator taking the value of 1 for children born to mothers under the age of 16 at the time of childbirth and 0 otherwise.
Outcomes	
Height-for-age (HAZ)	Standardized height indicator (z-scores) according to age- and gender-specific child growth references of a universally comparable well-nourished population (WHO, 2007). Values outside the -6:6 range are considered biologically implausible and set to missing.
Math score	Scores computed using Item Response Theory (IRT) models, which enhance score comparability over time and across ages. This score derives from a mathematical test covering a wide range of difficulty, from basic number identification and quantity discrimination, to calculation, measurement and items related to problem solving of real-life math applications.
Transmission channels	
Birthweight	Retrospectively collected in grams, from birth documents whenever possible.
Dietary diversity	Nutritional quality measure reflecting macro- and micronutrient adequacy of children. The construction of this variable follows the guidelines by Bilinsky and Swindale (2006) which suggests a 0-8 score. The data for this paper however allow for a 0-7 score. The indicator counts the number of nutritionally meaningful food groups consumed by the child in the previous 24 hours. The food groups are: grains, roots or tubers; vitamin A-rich plant foods, fruits and vegetables; meat, poultry, fish and seafood; eggs; pulses, legumes and nuts; milk and milk products; food items cooked in oil/fat.
Overage	Indicator taking the value of 1 if child is overaged for the school she is enrolled in at the beginning of the school year and 0 otherwise. The variable takes into account the official entrance age for each grade in the states of Andhra Pradesh and Telangana: age 3 for pre-primary education, 6 for primary education, 11 for upper primary education, 14 for high school, 16 for senior secondary and 18 for university. Only children in full-time enrolment are considered.
Education expenditure	Share of total household expenditure per capita in real terms assigned to educational fees, including school fees and private tuition fees. The consumer price index used as deflator to obtain real values (base year 2006) is built using information from the Young Lives community questionnaire. Only children in full-time enrolment are considered.
Teacher's name	Indicator taking the value of 1 if the mother knows the name of the offspring's teacher and 0 otherwise. Only children in full-time enrolment are considered.
Control variables	
Age	Age in years (reported in months).
Gender	Binary indicator taking the value of 1 for female and 0 for males.
Birth order	Constructed by comparing the ages of all the siblings living in the same household during any of the five round interviews of the Young Lives study.
Mother's height	Mother's height in cm.

Ethnicity/caste	Ethnicity/caste indicator with the following categories: Scheduled Caste, Scheduled Tribe, Backward Class, and other.
Rural/urban	Binary indicator taking the value of 1 if the household is located in an urban area and 0 if located in a rural area.
Total expenditure	Total household expenditure in per capita and real terms. The consumer price index used as deflator to obtain real values (base year 2006) is built using information from the Young Lives community questionnaire.
Wealth index	Composite continuous measure of living standards equally weighting subindices for housing quality, access to services and consumer durables (Briones 2017).
Number of household shocks	Number of household shocks suffered by the household in Rounds 1, 2 and 3. For the first round, shocks in the past four years are considered. For the second and third round, the shocks experienced between rounds are considered (four and three years, respectively). Shocks include natural disasters, significant changes in the economy or in state regulation, theft, significant house damages and changes in the family such as death/illness of parents (Briones 2018).
Older sibling	This variable indicates who of the two siblings in the pairs is older, in which case takes the value of 1, and 0 otherwise.

Table A3. Sample characteristics: all index children and index children with siblings

Sample:	All index children		Index children with siblings	
	Mean	N	Mean	N
Household characteristics				
Maternal age	22.62	6,015	22.37	4,604
Mother's education	4.08	5,634	4.12	4,570
Mother's height	151.43	5,742	151.41	4,564
Total expenditure	1,085.68	5,611	1,061.49	4,509
Wealth tertiles				
First wealth tertile	0.34	5,753	0.33	4,603
Second wealth tertile	0.33	5,753	0.33	4,603
Third wealth tertile	0.33	5,753	0.34	4,603
Urban	0.28	5,717	0.28	4,583
Region				
Coastal Andrah	0.35	5,706	0.34	4,583
Rayalaseema	0.29	5,706	0.29	4,583
Telangana	0.35	5,706	0.37	4,583
Ethnicity/caste				
Scheduled Caste	0.18	6,033	0.18	4,604
Scheduled Tribe	0.15	6,033	0.15	4,604
Backward Class	0.46	6,033	0.47	4,604
Other	0.21	6,033	0.20	4,604
Offspring characteristics				
Age	11.62	5,740	11.59	4,604
Female	0.46	6,033	0.45	4,604
Birth order				
Firstborn	0.38	6,033	0.36	4,604
Second born	0.38	6,033	0.40	4,604
Third born	0.15	6,033	0.16	4,604

Notes: Statistics correspond to child-round-level observations from Rounds 3 (2009), 4 (2013) and 5 (2016) for two samples. The first sample consists of all index children with available information on the corresponding variable. The second sample covers all index children with available information on age, gender, birth order, maternal age and HAZ or math data for him/her and the sibling. All time-variant variables (wealth tertiles, total expenditure, location-related variables, mother's education and age of the child) are measured in the three rounds. Maternal age at birth is defined as the difference between the child's age and mother's age. Mother's education consists of her highest completed grade. Mother's height is reported in cm and birthweight in grams. Total expenditure refers to household total monthly expenditure per capita in 2006 constant rupees. A composite wealth index was used for the estimation of the share of observations within each wealth tertile (see Briones (2017) for a detailed description). For the computation of birth order, the ages among siblings that lived in the Young Lives household during any of the five survey rounds were compared.

Figure A2. Age histogram by maternal age groups

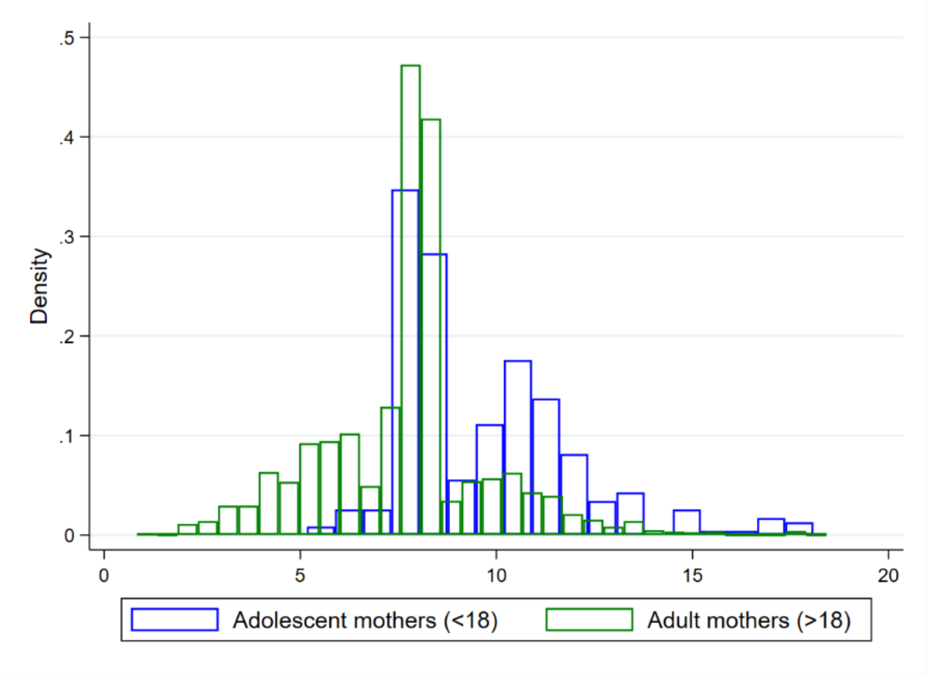

Notes: Histogram show the distribution of age of children at round 3. Sample refers to child-level observations with data on age, gender, birth order, maternal age and height for age (z-scores) or math (IRT scores) data. Adolescent mothers refers to children born to mothers under 18 years of age. Adult mothers refers to children born to mothers 18 years of age or older.

Figure A3. Falsification tests for HAZ

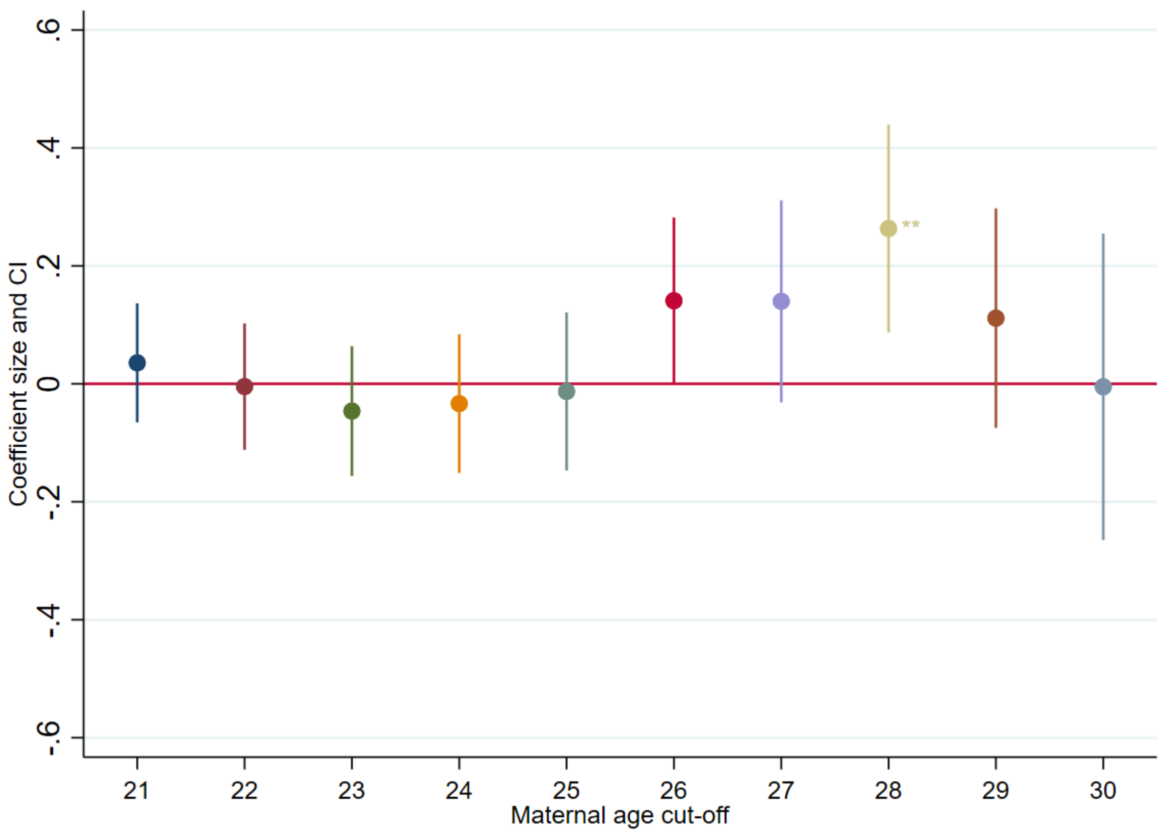

Notes: * p < 0.1; ** p < 0.05; *** p < 0.01. The sample includes sibling pairs for Rounds 3, 4 and 5. The dependent variable is HAZ (z-scores). For each coefficient estimation, the dependent variable is regressed on a binary indicator taking the value of 1 for children born to mothers under the specified maternal age cut-off and 0 otherwise. The included maternal age cut-offs have more than 60 households contributing to the coefficient of interest. All regressions control for mother fixed effects and for dummies for age, gender, birth order and round. The standard errors used for the estimation of the 90% confidence intervals (CI) are clustered at the mother level.

Table A4. Regression results: Older sibling, early childhood conditions and HAZ

	(1)<br>MFE	(2)<br>MFE	(3)<br>MFE	(4)<br>MFE	(5)<br>MFE	(6)<br>MFE
Panel A: HAZ						
Adolescent mother		-0.23***	-0.23***	-0.23***	-0.23***	-0.23***
		(0.08)	(0.08)	(0.08)	(0.08)	(0.08)
Older sibling	0.14	0.14				
	(0.10)	(0.10)				
Controlling for:						
Shocks			Yes			Yes
Wealth				Yes		Yes
Expenditure					Yes	Yes
R-squared	0.59	0.59	0.59	0.59	0.59	0.59
Observations	8698	8698	8631	8631	8631	8631
Panel B: Math						
Adolescent mother		-1.37	-1.55	-1.93	-1.44	-1.79
		(6.01)	(6.02)	(6.05)	(6.02)	(6.06)
Older sibling	13.64*	13.62*				
	(6.98)	(6.99)				
Controlling for:						
Shocks			Yes			Yes
Wealth				Yes		Yes
Expenditure					Yes	Yes
R-squared	0.68	0.68	0.68	0.68	0.68	0.69
Observations	5872	5872	5822	5822	5822	5822

Notes: * p < 0.1; ** p < 0.05; *** p < 0.01. Clustered standard errors at mother level in parentheses. The sample includes sibling pairs for rounds 3-5 in Panel A and rounds 4 and 5 in Panel B. Adolescent mother refers to children born to mothers under 18 years of age. Older sibling takes the value of 1 for older siblings and 0 for younger ones. The dependent variable is HAZ (z-scores) in Panel A and math (IRT scores) in Panel B. All regressions control for mother fixed effects and dummies for age, gender, birth order and round. The third, fourth and fifth column control for child-specific shocks, wealth index and real total expenditure per capita during early childhood, respectively. The sixth column controls for all three simultaneously.

Figure A4. Falsification tests for math

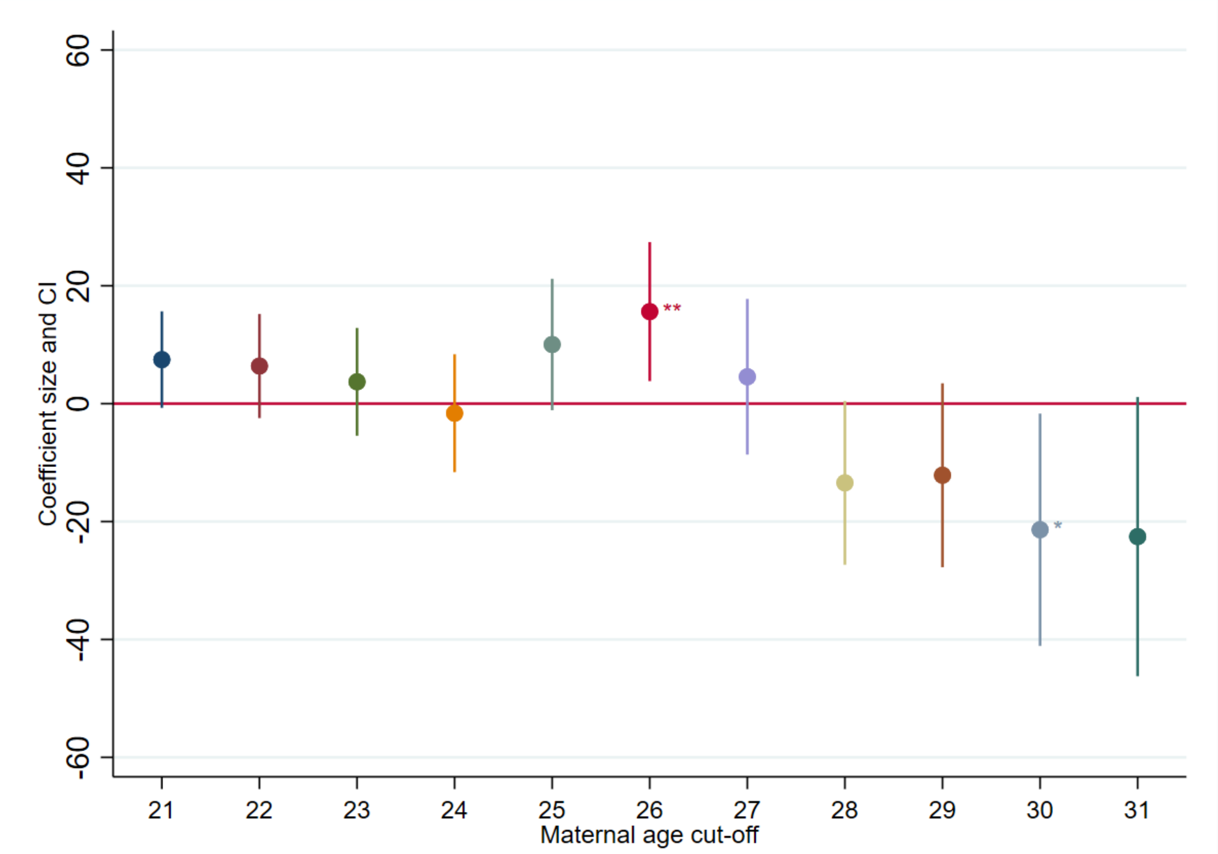

Notes: ***, **, * denote statistical significance at the 1, 5 and 10% level, respectively. The sample includes sibling pairs for Rounds 4 and 5. The dependent variable is math (IRT scores). For each coefficient estimation, the dependent variable is regressed on a binary indicator taking the value of 1 for children born to mothers under the specified maternal age cut-off and 0 otherwise. The included maternal age cut-offs have more than 60 households contributing to the coefficient of interest. All regressions control for mother fixed effects and for dummies for age, gender, birth order and round. The standard errors used for the estimation of the 90% confidence intervals (CI) are clustered at the mother level.

Table A5. Regression results: missing birthweight data

	Missing birthweight data	
	(1)	(2)
	OLS	MFE
Very young mother	-0.12**	-0.32***
	(0.05)	(0.09)
Young mother	0.02	-0.00
	(0.03)	(0.05)
R-squared	0.052	0.359
Observations	3364	3395

Notes: * p < 0.1; ** p < 0.05; *** p < 0.01. Clustered standard errors at mother level in parentheses. The sample consist of sibling pairs for which HAZ or math data are available. Very young mothers and young mothers refer to children born to mothers under 16 years of age and 16-17 years old, respectively. The reference category is the maternal age group of mothers 18 years old and older. The dependent variable is a binary indicator taking the value of 1 if birthweight data is missing and 0 otherwise. OLS regression controls for gender, birth order, ethnicity, rural/urban status in round 1 and mother's height. The MFE regression controls for mother fixed effects.
